# A compendium of geochemical information from the Saanich Inlet water column

**DOI:** 10.1038/sdata.2017.159

**Published:** 2017-10-31

**Authors:** Mónica Torres-Beltrán, Alyse K. Hawley, David Capelle, Elena Zaikova, David A. Walsh, Andreas Mueller, Melanie Scofield, Chris Payne, Larysa Pakhomova, Sam Kheirandish, Jan Finke, Maya Bhatia, Olena Shevchuk, Esther A. Gies, Diane Fairley, Céline Michiels, Curtis A. Suttle, Frank Whitney, Sean A. Crowe, Philippe D. Tortell, Steven J. Hallam

**Affiliations:** 1 Department of Microbiology and Immunology, University of British Columbia, Vancouver, British Columbia, Canada V6T 1Z3; 2 Earth, Ocean and Atmospheric Sciences, University of British Columbia, Vancouver, British Columbia, Canada V6T 1Z4; 3 Department of Biology, Georgetown University, Washington, DC 20007, USA; 4 Department of Biology, Concordia University, Montreal, Quebec, Canada H4B 1R6; 5 Department of Civil Engineering, University of British Columbia, Vancouver, BC, Canada V6T 1Z4; 6 Department of Botany, University of British Columbia, Vancouver, British Columbia, Canada V6T 1Z4; 7 Department of Fisheries and Oceans Canada, Sidney, British Columbia, Canada V9L 6V9; 8 ECOSCOPE Training Program, University of British Columbia, Vancouver, British Columbia, Canada V6T 1Z4; 9 Peter Wall Institute for Advanced Studies, University of British Columbia, Canada V6T 1Z2; 10 Genome Science and Technology Program, University of British Columbia, Vancouver, BC, Canada V6T 1Z4; 11 Graduate Program in Bioinformatics, University of British Columbia, Vancouver, British Columbia, Canada V6T 1Z4

**Keywords:** Marine chemistry, Microbial ecology

## Abstract

Extensive and expanding oxygen minimum zones (OMZs) exist at variable depths in coastal and open ocean waters. As oxygen levels decline, nutrients and energy are increasingly diverted away from higher trophic levels into microbial community metabolism, resulting in fixed nitrogen loss and production of climate active trace gases including nitrous oxide and methane. While ocean deoxygenation has been reported on a global scale, our understanding of OMZ biology and geochemistry is limited by a lack of time-resolved data sets. Here, we present a historical dataset of oxygen concentrations spanning fifty years and nine years of monthly geochemical time series observations in Saanich Inlet, a seasonally anoxic fjord on the coast of Vancouver Island, British Columbia, Canada that undergoes recurring changes in water column oxygenation status. This compendium provides a unique geochemical framework for evaluating long-term trends in biogeochemical cycling in OMZ waters.

## Background & Summary

Marine oxygen minimum zones (OMZs) are widespread, naturally occurring water column features that arise from respiration of organic matter in subsurface waters with restricted circulation. Operationally defined by oxygen (O_2_) concentrations between 0 to 20 μM, and differential accumulation of nitrite (NO_2_^−^) and reduced sulphur compounds, OMZs currently constitute 1–7% of global ocean volume^1–7^. As oxygen levels decline, nutrients and energy are increasingly diverted away from higher trophic levels into microbial community metabolism^2,8^. As a result, OMZs are hotspots for the biogeochemical cycling of carbon, nitrogen and sulphur with resulting feedback on nitrogen loss processes and climate active trace gas production including nitrous oxide (N_2_O) and methane (CH_4_)^9–13^. The effects of climate change, including increased stratification and reduced O_2_-solubility in warming waters are resulting in OMZ expansion and intensification^1,8,14–18^ reinforcing the need to monitor changes in water column geochemistry in oxygen-deficient waters.

Oceanographic surveys in OMZ waters rely on a standard suite of measurements including temperature, salinity, density and conductivity. Additional parameters including irradiance, used to measure water column light penetration, fluorescence used to monitor chlorophyll concentrations and dissolved gases including O_2_ and carbon dioxide (CO_2_) provide information on primary production^2,19,20^. Chemical measurements of phosphate (PO_4_^3−^), silicic acid (SiO_2_), and nitrate (NO_3_^−^) are measured as essential nutrients supporting growth and cell division^15^. Nitrite (NO_2_^−^) and ammonium (NH_4_^+^) are also measured to better constrain nitrogen cycling processes^2,10,11,13^. Because some OMZs can become completely anoxic, hydrogen sulfide (H_2_S) concentrations can be used as an indicator for sulphate reduction driving chemoautotrophic metabolism^3,9^. Measurements of N_2_O and CH_4_ can also be used to monitor potential climatological impacts of OMZ expansion^9–13,21^. Collectively, these measurements define geochemical gradients in OMZ water columns that shape the conditions for coupled biogeochemical cycling.

Saanich Inlet is a seasonally anoxic fjord on the coast of Vancouver Island, British Columbia, Canada^22–25^. Saanich Inlet is an inverse estuary where a glacial sill at the mouth restricts exchange between deep basin and external waters for most of the year. Freshwater is supplied at the inlet mouth predominantly by the Cowichan and Fraser Rivers, producing horizontal density differences that result in an inward flow into the inlet in the surface layer and outward flow at depth^25,26^. During spring and summer months, high levels of primary productivity in surface waters and limited vertical mixing of basin waters below the sill result in anoxia and the accumulation of CH_4_, NH_4_^+^ and H_2_S^27–29^. In late summer and fall, neap tidal flows produce an influx of denser water from the Northeastern subarctic Pacific (NESAP) Ocean that cascade over the sill, resulting in vertical mixing and the re-supplying of deep basin waters with O_2_ and nutrients^25,26^. The recurring seasonal development of water column anoxia followed by deep water renewal makes Saanich Inlet a model ecosystem for monitoring biogeochemical responses to changing levels of water column O_2_-deficiency^2,30–32^.

**Figure 1:**
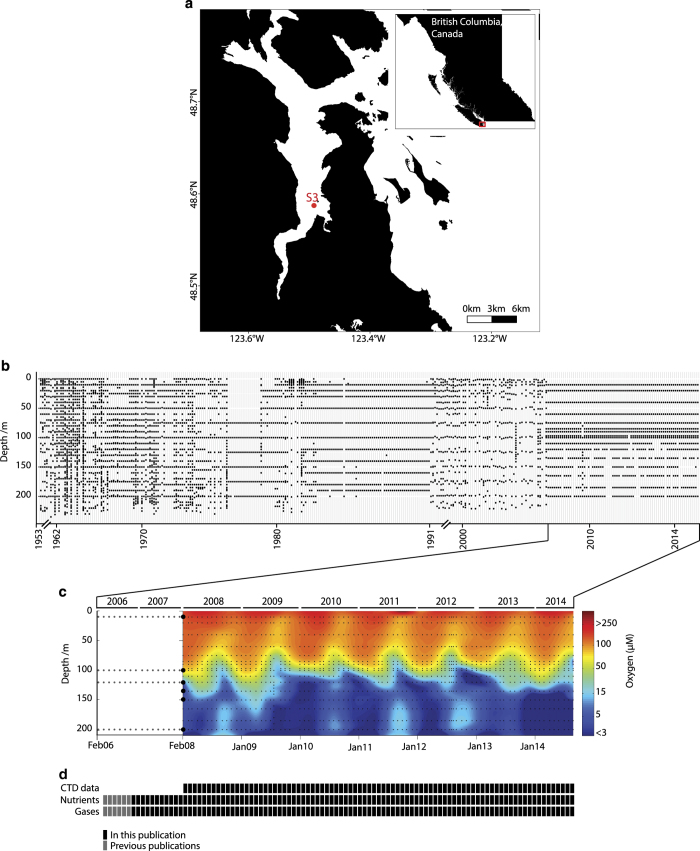
Geochemical data time series in the Saanich Inlet. (**a**) Sampling station S3 location in the Saanich Inlet. (**b**) Historical sampling effort in Saanich Inlet depicted as O_2_ sampling points from 1953 to 2014. (**c**) Oxygen concentration contour for CTD data (February 2008 onward), and points for 16 sampling depths for nutrients and gases. (**d**) Sample inventory from February 2006 to October 2014 showing historical, CTD and nutrient datasets included in this manuscript (solid black), in previous publications (gray).

Here we present a compendium of time-series observations encompassing historical O_2_ measurements^25,33^ (Fig. 1a) and more recent monthly monitoring efforts in Saanich Inlet from 2006 through 2014, representing over 100 independent sampling expeditions (Fig. 1b). This compendium contains physical (temperature, salinity, density, irradiance, and fluorescence), chemical (PO_4_^3−^, SiO_2,_ NO_3_^−^, NO_2_^−^, NH_4_^+^, and H_2_S), dissolved gas (O_2_, CO_2_, N_2_, N_2_O, CH_4_), and biological (cell counts) parameter data (Fig. 1b,c) useful in comparing to other oceanographic time-series from the Northwest Atlantic to Eastern Tropical Pacific through the Global Ocean Sampling expeditions^34^, the Hawaii and Tara Oceans^34–36^ and Bermuda Atlantic Time-series^37^ and in the development of biogeochemical models. In addition, this geochemical compendium is paired with a cognate compendium of multi-omic sequence information (DNA, RNA, protein) focused on microbial diversity, abundance and function.^38^ Combined, these compendiums provide a community-driven framework for observing and predicting microbial community repsonses to changing levels of oxygen deficiency extensible to open ocean OMZs.

## Methods

Time-series monitoring in Saanich Inlet was conducted on a monthly basis aboard the *MSV John Strickland* at station S3 (48°35.500 N, 123°30.300 W) as previously described^32^. Water samples from 16 high-resolution (HR) depths at station S3 (10, 20, 40, 60, 75, 80, 90, 97, 100, 110, 120, 135, 150, 165, 185 and 200 meters) spanning oxic (>90 μmol O_2_ kg^−1^), dysoxic (90–20 μmol O_2_ kg^−1^), suboxic (20–1 μmol O_2_ kg^−1^) anoxic (<1 μmol O_2_ kg^−1^) and sulfidic water column compartments^2^ were collected using Niskin or Go-Flow bottles for dissolved gasses: O_2_, CO_2_, CH_4_, Nitrogen gas (N_2_), N_2_O; nutrients: NO_3_^−^, NO_2_^−^, NH_4_^+^, SiO_2_, PO_4_^3−^, H_2_S; and cell counts. Sampling methods for HR samples and additional six large-volume depths (10, 100, 120, 135, 150 and 200 meters) collected for time-series multi-omic sequence information analyses are published in an accompanying compendium^38^.

### Environmental sampling

**Figure 2:**
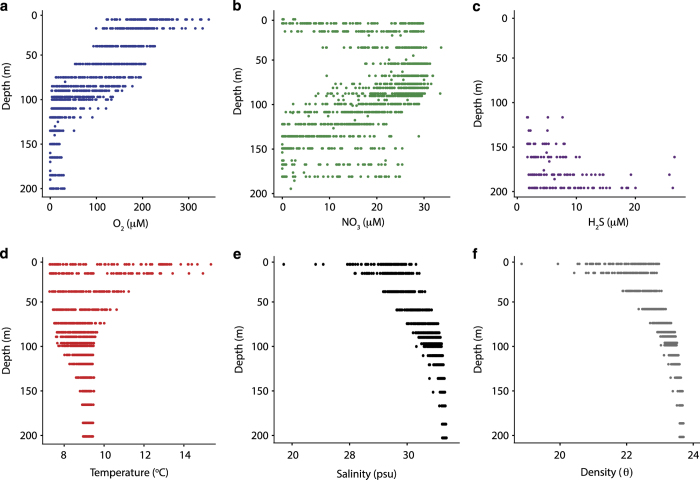
Time series environmental parameters water column profiles. Panel showing dot plots for oxygen (O_2_; blue), nitrate (NO_3_^−^; green), hydrogen sulphide (H_2_S; purple), temperature (°C; red), salinity (psu; black) and density (θ; gray) measurements along the depth profile for samples taken from February 2008 to October 2014 at Station S3 in Saanich Inlet.

Historical dissolved O_2_ concentrations were obtained from station S3 by sampling with Niskin bottles at discrete depths and subsequently analyzing water samples using various modifications of the Winkler method^25,33,39^ (Data Citation 1). Historical water column profiles can also be accessed at the Ocean Sciences Data Inventory website hosted by the Institute of Ocean Sciences and Fisheries and Oceans Canada (http://www.pac.dfo-mpo.gc.ca/science/oceans/data-donnees/search-recherche/profiles-eng.asp). Samples collected from February 2006 to February 2008 were processed and analysed for dissolved gases and nutrients as first reported in Zaikova *et al.*^32^ (Fig. 2). Beginning on February 2008, a Sea-Bird SBE 25 CTD (conductivity, temperature and depth), with Sea-Bird SBE 43 dissolved O_2_ and Biospherical Instruments PAR sensors attached was used to measure conductivity, temperature, dissolved O_2_, PAR/Irradiance and fluorescence (Data Citation 1). To minimize the effects of off-gassing, waters were collected in the following order; dissolved O_2_ for Winkler titration (from select depths for CTD calibration), dissolved gases (N_2_O and CH_4_), NH_4_^+^, H_2_S, nutrients, cell counts (Data Citation 1) and salinity (from selected depths for CTD calibration). A detailed seawater sampling video protocol can be found online (http://www.jove.com/video/1159/seawater-sampling-and-collection).

### Chemical data

#### CTD data analysis

CTD data were downloaded, converted and pre-processed in the laboratory using the SeaBirdSeasoft software. Downcast data of the deepest cast (200 m) was extracted and converted from ASCII format into a.cnv file for manual curation. Salinity and density were calculated using the Derive module with the corrected conductivity measurements. Temperature and salinity were exported using an ITS-90 scale. Oxygen sensor measurements collected in millilitre per litre (mll^−1^) were converted to micromolar (μM) units (Data Citation 1). Discrete winkler analyses from water samples spanning LV depths were used to calibrate the CTD O_2_ measurements (Data Citation 1).

#### Nitrate, phosphate and silicic acid

For each depth, sample water was filtered through a 0.2 μm acrodisc (Millipore) and used to rinse a 15 ml tube three times before filling with 14 ml. Samples were stored on ice and later in the lab at −20 °C for up to four months prior to analysis. A Bran Luebbe AutoAnalyser 3 using air-segmented continuous-flow and standard colorimetric methods was used for analysis. In brief, nitrate (NO_3_^−^) was reduced to nitrite by a copper-cadmium reduction column. Nitrite was then quantified by a modified colorimetric assay^40^, reading sample absorbance at 550 nm. Orthophosphate (PO_4_^3−^) was quantified based on the colorimetric method for reduced phospho-molybdenum complex, reading samples absorbance at 880 nm^41^. Silicic acid (H_4_SiO_4_) was quantified by reduction to a molybdenum blue complex, reading sample absorbance at 820 nm. Oxalic acid was added to remove phosphate interference^40^ (Data Citation 1).

#### Ammonium

**Figure 3:**
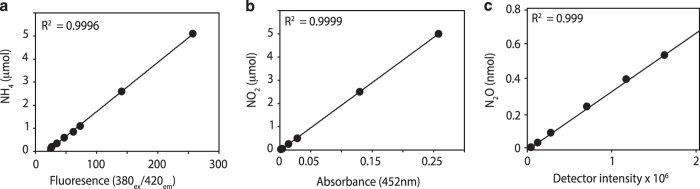
Validation for environmental parameters. (**a**–**c**) Typical standard curves for chemical parameters ammonium (**a**) and nitrite (**b**), gas concentration standard curves for Nitrous oxide (**c**).

A fluorometric measurement protocol for NH_4_^+^ analysis was carried out as previously described in Holmes *et al.* for marine samples^42^. For each depth, glass amber scintillation bottles were rinsed three times, then filled to overflowing and capped immediately to minimize off-gassing of NH_4_^+^ and stored on ice for 1–3 h before processing. A total of 5 ml of sample water was transferred to vials with 7.5 ml o-phthaldialdehyde (OPA; Sigma) in triplicate. Simultaneously, 7.5 ml of OPA was added to prepared NH_4_^+^ standard curve (0.025–10.0 μM NH_4_Cl) and stored at room temperature for up to 4 h. Fluorescence at 380_ex_/420_emm_ was read using a Turner Designs TD-700 fluorometer (2006–2009) or Varioskan plate reader (2009–2014) in triplicate with 300 μl of sample or standard in a 96-well round bottom plate (Corning) (Fig. 3) (Data Citation 1).

#### Nitrite

The protocol for NO_2_^−^ analysis was carried out as previously described in Armstrong *et al.* modified for marine samples^40^. For each depth, sample water was filtered through a 0.2 μm acrodisc (Millipore) and used to rinse a 15 ml tube three times before filling with 14 ml filtered sample water and stored on ice for 1–3 h before processing. A total of 2 ml of sample water was transferred to 4 ml plastic cuvettes in triplicate, and 100 μl sulphanilamide and 100 μl nicotinamide adenine dinucleotide (NAD) were added. Simultaneously, reagents were added to prepared standards (0.025–5.0 μM NaNO_2_). Cuvettes were inverted and stored on ice for up to 4 h. Absorbance at 542 nm was read using a Cary60 spectrometer (Fig. 3) (Data Citation 1).

#### Hydrogen sulfide

The protocol for H_2_S was carried out as previously described in Cline^43^ modified for marine samples. For each depth, 10 ml sample water was collected directly into a 15 ml tube containing 200 μl 20% Zinc Acetate and stored on ice for 4–24 h before processing. A total of 300 μl of sample was transferred into triplicate wells of a 96-well round or flat-bottom plate (Corning), and 6 μl Hach Reagent (Hach) 1 and 2 for sulphide assay were added to each well. After 5 min incubation, absorbance at 670 nm was read using a spectrophotometer (2008–2009) or Varioskan plate reader (2009–2014) (Data Citation 1).

#### Cell counts

For each depth, 10 ml sample water was collected directly into a sterile 15 ml tube containing 1.1 ml of 37% formaldehyde and stored on ice. Back at the lab, samples were stored at 4 °C for up to two days prior to cell counting using a BD LSR II flow cytometer (2008–2012) or MACS Quant Analyzer (2012–2014) based on the following protocols. For BD LSR II, a dye mixture was prepared by diluting 3 μl of the SYBR Green I (Invitrogen) dye in 1,830 μl of sterile water. Six drops (Alignflow) alignment beads were then added to this mixture. In a round-bottom polystyrene tubes, 25 μl of the dye mix was added to 475 μl of the water sample (in triplicates). The cells and beads were then counted using BD LSR II flow cytometer. For MAXSQuant, a dye mixture was prepared by diluting 240 μl of seawater sample with 10 μl of SYBR Green I (Invitrogen) dye mix which contains 6 μm flow cytometry blue laser alignment beads (Alignflow), for calibration purposes. SYBR Green mix was prepared by diluting 4 μl of the dye in 1,570 μl of sterile water following an addition of 30 μl beads. Samples are prepared in triplicates in a 96-well flat bottom black plate (Corning) and run on MACSQuant Analyzer (MiltenyiBiotec) (Data Citation 1).

#### Dissolved gases

For each depth, sample water was collected through silicon tubing (~15 cm long and 1/4″ thick, pre-flushed for a few seconds with sample water) into a 30 or 60 ml borosilicate glass serum vial, overflowing three times the volume and taking care to remove air bubbles from the tubing and vial during filling. The vials were spiked with 50 μl saturated mercuric-chloride solution, then crimp-sealed with a butyl-rubber stopper and aluminium cap. Samples were stored in the dark at 4 °C until processing. Dissolved gases were analysed using either headspace for CO_2_, CH_4_, N_2_ and N_2_O (2006–2009, samples stored for up to 2 years) or automated purge-and-trap for CH_4_ and N_2_O only (2009–2014, samples stored for <3 months) coupled with gas chromatography-mass spectrometry (GC-MS)^44^ (Data Citation 1). Samples with >20% s.d. between replicates were excluded from our study to discard any long storage effects.

## Data Records

### Data record 1

The Saanich Inlet O_2_ historical data (1953–2000) is accessible in comma-separated-value format file ‘Historical_O2_DATA.csv’ on the Dryad Digital Repository [Data Citation 1] containing the data fields outlined in Table 1.

### Data record 2

The Saanich Inlet time-series CTD data is accessible in comma-separated-value format file ‘Saanich_TimeSeries_CTD_DATA.csv’ on the Dryad Digital Repository [Data Citation 1] domain containing the data fields outlined in Table 2.

### Data record 3

The Saanich Inlet time-series chemical data is accessible in comma-separated-value format file ‘Saanich_TimeSeries_Chemical_DATA.csv’ on the Dryad Digital Repository domain containing the data fields outlined in Table 3 [Data Citation 1].

### Data record 4

The Saanich Inlet time-series Winkler O_2_ data is accessible in comma-separated-value format file ‘Saanich_TimeSeries_Winkler_DATA.csv’ on the Dryad Digital Repository [Data Citation 1] domain containing the data fields outlined in Table 4.

## Technical Validation

### Data quality control

Data in the Saanich Inlet time series was collected and processed by experienced scientists with extensive training in the sampling methods and data processing steps described above. People interested in becoming part of the scientific crew were invited to participate in training sessions with experienced scientists in the field and laboratory to gain practical experience. Once in the field, trainees were carefully supervised during sample collection for a minimum of 3 months for quality assurance. Following each cruise, the acting chief scientist compiled all chemical and physical data collected and conducted initial quality controls, checking for outliers and verifying standard curves. Data were then entered into an in-house database along with field notes and precise records of volumes of water filtered informing downstream analyses.

### CTD and chemical data validation

The SeaBird 43 dissolved O_2_ sensor was calibrated by Winkler O_2_ measurements^45^. Samples from selected depths were collected into Winkler glass Erlenmeyer flasks using latex tubing, overflowing three times to ensure no air contamination. Oxygen concentration was determined using a Brinkman autotitrator, routinely calibrated with a potassium iodide standard. Stability of CTD O_2_ measurements was determined by comparing the high values with Winkler measurements, and low values with sulfidic profiles where the sensors levels off. Where H_2_S is detected we consider O_2_ measurements to be 0 μM based on spontaneous auto-oxidation reaction of H_2_S with O_2_. We have estimated our limit of detection for the automated Winkler method at ~0.007 mll^−1^ or ~0.3 μM.

The SeaBird conductivity sensor was calibrated using salinity samples collected at selected depths. Salinity glass bottles were rinsed 4 times and filled with water sample, stored at room temperature and analyzed within 4 months on a Guildline Portasal salinometer.

For each cruise, standard curves for NH_4_^+^ and NO_2_^−^ were prepared. Stock solutions and reagents for both assays were freshly made every three months and stored in the dark at 4 °C and were tested prior to being used for analysis. Stock solution quality and assay validation was carried out using linear regression and calculating the r squared value (r^2^≥0.90) on the absorbance data (Fig. 3). Standard curve stock solutions and reagents for H_2_S assay were evaluated every three months based on manufacturer’s instructions. We have estimated our limit of detection for these assays to be 0.001 μM NH_4_^+^, 0.0006 μM NO_2_^−^, and 1.7 μM H_2_S.

Samples for NO_3_^−^, PO_4_^3−^ and H_4_SiO_4_ were run in single measurements. Autoanalyzer estimated limit of detection for these measurements are 0.020 μM NO_3_^−^, 0.012 μM PO_4_^3−^ and 0.100 μM H_4_SiO_4_.

### Flow cytometry validation

Concentration of flow cytometry (FL) alignment beads was determined by microscopy using a hemocytometer. Bead counts for each FL run were then used to calculate the volume of sample measured. Two blanks were included in each FL run, and consisted of sterile water bead/dye solution with sterile water in place of sample water, to ensure instrument cleanliness and optics function. Size gates were set to include beads and bacterial and archaeal cell sizes and to reduce noise of any small particulate debris.

### Gas analysis validation

A thorough review of the Purge and Trap GCMS (PT-GCMS) method validation has been previously described^44^. Standard curves were run at the start of each batch of 25 samples by injecting precisely measured quantities of a standard gas mixture (CH_4_, N_2_O, CO_2_ and N_2_) calibrated against National Ocean and Atmospheric Administration (NOAA) certified reference gas mixture. Single standards were also measured every ~2-hours (5–6 sample per run) to monitor instrument drift. The precision of CH_4_ and N_2_O measurements based on replicate measurements of air-equilibrated water samples was <4%. Accuracy was confirmed by measuring dissolved N_2_O and CH_4_ in carefully prepared air-equilibrated, temperature-controlled Milli-Q water and comparing this to expected concentrations based on gas-solubility equations^46,47^. Detection limits depend on the volume of sample being purged, and were 0.8 nM for CH_4_ and 0.5 nM for N_2_O for the samples analyzed in this time-series (2009–2014) (Fig. 3). Samples were run in duplicate or triplicate to ensure reproducible readings. The relative s.d. between replicate samples was calculated and included in the output data. The output data are also carefully inspected to ensure optimal instrument performance during sample analysis before being submitted to the database.

## Usage Notes

### Oxygen considerations

Based on the amount of dissolved O_2_ in the water column and the biogeochemical processes associated with it, thresholds for O_2_-defined water column conditions were determined as previously described^2^. As the range of O_2_ concentrations is wide and has great impact on biological processes in the lower concentrations, we suggest using the O_2_ thresholds described in Wright *et al.*^2^ for analysis.

## Additional information

**How to cite this article:** Torres-Beltrán, M. *et al.* A compendium of geochemical information from the Saanich Inlet water column. *Sci. Data* 4:170159 doi: 10.1038/sdata.2017.159 (2017).

**Publisher’s note:** Springer Nature remains neutral with regard to jurisdictional claims in published maps and institutional affiliations.

## Supplementary Material



## Figures and Tables

**Table 1:** Key to the data fields in the Saanich Inlet historical O_2_ dataset.

**Data field**	**Description**	**Units**
*Longitude*	Unique geographical coordinates for sampling station	Decimal degrees followed by letter
*Latitude*	Unique geographical coordinates for sampling station	Decimal degrees followed by letter
*Date*	Year of cruise	Numeric string as YYYY-M/YYYY-MM-DD
*Depth*	Depth measurement in different intervals	Meters (m)
*Temperature*	Temperature measurement at each pressure point	Celsius degrees (°C)
*Salinity*	Salinity measurement at each depth	Practical salinity unit (PSU)
*Density*	Density measurement at each depth	Sigma-theta (q)
*Oxygen (ml)*	Dissolved oxygen concentration at each depth	Millilitres per liter (mll^−1^)
*Oxygen (μM)*	Dissolved oxygen concentration at each depth	Micromolar (μM)

**Table 2:** Key to the data fields in the Saanich Inlet time-series CTD dataset.

**Data field**	**Description**	**Units**
*Longitude*	Unique geographical coordinates for sampling station	Decimal degrees followed by letter
*Latitude*	Unique geographical coordinates for sampling station	Decimal degrees followed by letter
*Cruise number*	Numerical identifier of individual cruises	Numeric string
*Date*	Year of cruise	Numeric string as YY-MM-DD
*Cruise day*	Day of cruise	Numeric string
*Pressure*	CTD pressure measurement in intervals of 1 meter	Decibars (db)
*Temperature*	CTD temperature measurement at each pressure point	Celsius degrees (°C)
*Conductivity*	CTD conductivity sensor measurement at each pressure point	Millisiemens per centimetre (mScm^−1^)
*Fluorescence*	CTD Wetstar fluorometer chlorophyll measurement at each pressure point	Chlorophyll concentration in milligram per cubic meter (mgm^−3^)
*Beam transmission*	CTD transmissometer measurement at each pressure point	Light transmission (%)
*PAR/Irradiance*	CTD Photosintentically active radiation (PAR) measurement at each pressure point	Irradiance
*Oxygen SBE*	CTD Dissolved oxygen sensor measurement at each pressure point	Volts (V)
*Oxygen (μM)*	Oxygen calculation based on CTD Oxygen SBE following this calculation: [µmoleperKg]=SBE*×44660/(sigma−theta+1000) *where:—*44660 constant for oxygen gas:—Sigma-theta is the density	Micromolar (μM)
*Salinity*	CTD salinity measurement at each pressure point	Practical salinity unit (PSU)
*Density*	CTD density measurement at each pressure point	Sigma-theta (q)

**Table 3:** Key to the data fields in the Saanich Inlet time-series chemical dataset.

**Data field**	**Description**	**Units**
*Longitude*	Unique geographical coordinates for sampling station	Decimal degrees followed by letter
*Latitude*	Unique geographical coordinates for sampling station	Decimal degrees followed by letter
*Cruise*	Numerical identifier of individual cruises	Numeric string
*Date*	Year of cruise	Numeric string as YY-MM-DD
*Depth*	Sampling depth	Meters (m)
*Ctd_O*_*2*_	Oxygen concentration calculated from CTD, as indicated in Table 1, for each depth	Micromolar (μM)
*PO_4_*	Phosphate concentration for each depth	Micromolar (μM)
*SiO*_*2*_	Silicate concentration for each depth	Micromolar (μM)
*NO*_*3*_	Nitrate concentration for each depth	Micromolar (μM)
*Mean_NH*_*4*_	Average concentration of Ammonium for each depth	Micromolar (μM)
*Std_NH*_*4*_	Standard deviation for Ammonium average concentration	Micromolar (μM)
*Mean_NO*_*2*_	Average concentration of Nitrite for each depth	Micromolar (μM)
*Std_NO*_*2*_	Standard deviation for Nitrite average concentration	
*Mean_H*_*2*_*S*	Average concentration of Hydrogen sulfide for each depth	Micromolar (μM)
*Std_H*_*2*_*S*	Standard deviation for Hydrogen sulfide average concentration	
*Cells/ml*	Cell counts value quantified by flow cytometry	Number of cells per millilitre (cells per ml)
*Mean_N*_*2*_	Average concentration of Nitrogen gas for each depth	Micromolar (μM)
*Std_N*_*2*_	Standard deviation for Nitrogen gas average concentration	
*Mean_O*_*2*_	Average concentration of Oxygen for each depth	Micromolar (μM)
*Std_O*_*2*_	Standard deviation for Oxygen average concentration	
*Mean_CO*_*2*_	Average concentration of Carbon dioxide for each depth	Micromolar (μM)
*Std_CO*_*2*_	Standard deviation for Carbon dioxide average concentration	
*Mean_N*_*2*_*O*	Average concentration of Nitrous oxide for each depth	Nanomolar (μM)
*Std_N*_*2*_*O*	Standard deviation for Nitrous oxide average concentration	
*Mean_CH*_*4*_	Average concentration of Methane for each depth	Nanomolar (μM)
*Std_CH*_*4*_	Standard deviation for Methane average concentration	

**Table 4:** Key to the data fields in the Saanich Inlet Winkler O_2_ dataset.

**Data field**	**Description**	**Units**
*Longitude*	Unique geographical coordinates for sampling station	Decimal degrees followed by letter
*Latitude*	Unique geographical coordinates for sampling station	Decimal degrees followed by letter
*Cruise number*	Numerical identifier of individual cruises	Numeric string
*Date*	Year of cruise	Numeric string as YY-MM-DD
*Depth*	Depth measurement in different intervals	Meters (m)
*Oxygen*	Dissolved oxygen concentration at each depth measured by Winkler method	Millilitres per liter (mll^−1^)
