# Author Correction: A compendium of geochemical information from the Saanich Inlet water column

**DOI:** 10.1038/s41597-018-0005-2

**Published:** 2019-01-15

**Authors:** Mónica Torres-Beltrán, Alyse K. Hawley, David Capelle, Elena Zaikova, David A. Walsh, Andreas Mueller, Melanie Scofield, Chris Payne, Larysa Pakhomova, Sam Kheirandish, Jan Finke, Maya Bhatia, Olena Shevchuk, Esther A. Gies, Diane Fairley, Céline Michiels, Curtis A. Suttle, Frank Whitney, Sean A. Crowe, Philippe D. Tortell, Steven J. Hallam

**Affiliations:** 1 Department of Microbiology and Immunology, University of British Columbia, Vancouver, British Columbia V6T 1Z3 Canada; 2 Earth, Ocean and Atmospheric Sciences, University of British Columbia, Vancouver, British Columbia V6T 1Z4 Canada; 3 Department of Biology, Georgetown University, Washington, DC 20007 USA; 4 Department of Biology, Concordia University, Montreal, Quebec, H4B 1R6 Canada; 5 Department of Civil Engineering, University of British Columbia, Vancouver, BC V6T 1Z4 Canada; 6 Department of Botany, University of British Columbia, Vancouver, British Columbia V6T 1Z4 Canada; 7 Department of Fisheries and Oceans Canada, Sidney, British Columbia V9L 6V9 Canada; 8 ECOSCOPE Training Program, University of British Columbia, Vancouver, British Columbia V6T 1Z4 Canada; 9 Peter Wall Institute for Advanced Studies, University of British Columbia, British Columbia, V6T 1Z2 Canada; 10 Genome Science and Technology Program, University of British Columbia, Vancouver, BC V6T 1Z4 Canada; 11 Graduate Program in Bioinformatics, University of British Columbia, Vancouver, British Columbia V6T 1Z4 Canada

Correction to: *Scientific Data* 10.1038/sdata.2017.159, published online 31 October 2017

In Table 3 of this Data Descriptor the units of *Mean_N*_*2*_*O* and *Mean_CH*_*4*_ are incorrectly stated as “Nanomolar (μM)”. This should instead read “Nanomolar (nM)”.

